# Response to Three Weeks of Sprint Interval Training Cannot Be Explained by the Exertional Level

**DOI:** 10.3390/medicina56080395

**Published:** 2020-08-07

**Authors:** Raulas Krusnauskas, Nerijus Eimantas, Neringa Baranauskiene, Tomas Venckunas, Audrius Snieckus, Marius Brazaitis, Hakan Westerblad, Sigitas Kamandulis

**Affiliations:** 1 Institute of Sport Science and Innovations, Lithuanian Sports University, 44221 Kaunas, Lithuania; Nerijus.Eimantas@lsu.lt (N.E.); Neringa.Baranauskiene@lsu.lt (N.B.); Tomas.Venckunas@lsu.lt (T.V.); Audrius.Snieckus@lsu.lt (A.S.); Marius.Brazaitis@lsu.lt (M.B.); Hakan.Westerblad@ki.se (H.W.); Sigitas.Kamandulis@lsu.lt (S.K.); 2 Department of Physiology and Pharmacology, Karolinska Institutet, 17177 Stockholm, Sweden

**Keywords:** aerobic capacity, anaerobic power, high-intensity interval training, high responder, low responder

## Abstract

*Background and Objectives:* The all-out mode of sprint interval training (SIT) has been shown to be an efficient method for improving sports performance, exercise capacity, and aerobic fitness. Although the benefits of SIT are well described, the mechanisms underlying the different degrees of response remain largely unexplored. We aimed to assess the effects of exertion on the responsiveness to SIT. *Materials and Methods:* The participants were 28 young untrained men (mean ± SD age 25.7 ± 6.03 years) who exhibited either a large or small increase in Wingate test average power in response to nine SIT sessions performed over three weeks. Each training session comprised four–six bouts of 30 s all-out cycling interspaced with 4 min of rest. Individual responses were assessed using heart rate (HR) during exercise for all nine sessions, as well as blood lactate concentration up to 1 h, and the decrement in maximal voluntary knee extension torque (MVC) up to 24 h after the first and last training sessions. Peak oxygen uptake (VO_2_peak) and maximum HR were measured before and after training during an incremental cycling test to exhaustion. *Results:* Although all participants showed benefits of SIT such as increased VO_2_peak, the increase in anaerobic cycling power varied between participants. We identified 17 high responders and nine low responders, whose average power outputs were 0.80 ± 0.22 and 0.22 ± 0.19 W/kg, respectively. The HR achieved during any of the training sessions did not differ between high and low responders. The lactate kinetics did not differ between groups before and after the intervention. Training resulted in a more rapid recovery of MVC without any discernible differences between the high and low responders. *Conclusion:* The differences in the responses to SIT are not dependent on the exertion level during training.

## 1. Introduction

Regular physical activity has significant benefits for exercise capacity and overall health [1], and much attention has focused on finding the most efficacious exercise training mode to improve various bodily functions. However, a sizable proportion of the population fails to meet the recommended physical activity guidelines despite the variety of efficient training regimes recommended, such as resistance training to improve muscle mass and strength, and endurance training to improve aerobic capacity and reduce the risk of obesity [2]. Recognition of this overall poor adherence to regular physical activity has shifted research toward exercise adherence rather than the efficiency of existing or new training modalities [3]. One possible explanation for the poor exercise adherence is the minimal improvements in exercise capacity or health markers in a proportion of the population labeled as low responders or non-responders [4,5]. Better understanding of the factors and mechanisms that contribute to exercise responsiveness may lead to better individualization of exercise prescription to maximize fitness gains and minimize the chance of a low adaptative response.

Currently, one the most studied exercise modes is high-intensity interval training (HIIT). Much attention is being given to the factors and mechanisms that influence the responsiveness to HIIT, especially one of its most effective modes, namely sprint interval training (SIT). SIT comprises short, maximal-effort activity bursts interspaced with passive to low exercise intensity recovery periods. The fast and efficient gains in athletic performance in response to SIT are well established and are considered to exceed those of traditional aerobic training as advocated over the past decades [6,7,8]. SIT also affects mood positively [9], improves cognition [10] and body composition [8,11], augments cardiac function [12], reduces markers of systemic inflammation, and improves quality of life [12,13,14]. These benefits make SIT an attractive time-saving training regimen for improving and maintaining physical health for professional athletes as well as many other populations.

We have observed that the exertion level during SIT sessions differs between young and older people [10,15] and that this difference may result in less exercise-induced stress, which may preclude full-scale adaptations in older people. Other populations may also exhibit considerable differences in exertion level. Very intense exercise such as SIT causes metabolic perturbations associated with unpleasant sensations [16]. To avoid this discomfort and perhaps the delayed side effects [17], some exercisers may reduce their effort, especially when SIT is implemented as a long-term training routine.

The aim of the current study was to test whether the level of exertion, measured as physiological stress markers during nine SIT sessions performed over three weeks of training, would influence the gains in exercise performance. We hypothesized that high responders to SIT would demonstrate higher levels of exertion compared with low responders.

## 2. Materials and Methods

### 2.1. Participants

Only 18–35-year-old men who did not perform any aerobic or strength training for the 6 months before the study were considered as potential participants. A total of 28 volunteers were enrolled after their health status had been checked by a medical doctor. The exclusion criteria were the presence of overt cardiovascular, pulmonary, or metabolic disease, or chronic joint pain. During the study, participants were not allowed to take part in any other structured physical activity except for low-intensity daily chores. Participants were instructed not to change their diet or start any new physical activities during the study. Before any study-related procedure, all participants read and voluntarily signed an informed consent form, which was consistent with the principles outlined in the Declaration of Helsinki. All procedures were approved by the Kaunas Regional Biomedical Research Ethics Committee.

### 2.2. Group Classification

Participants were classified into the high- and low-responder groups based on their individual change in average power between the first and last sessions and 2 × TE threshold-based dichotomous classification, as follows [5,18]:

TE = SD_(last – first)_/square root of 2

where TE is the typical error and SD is the standard deviation. This classification resulted in 17 high responders and nine low responders. Two participants were excluded because they fell within the “gray zone” of an uncertain response. The characteristics of the high and low responders are presented in Table 1.

### 2.3. Organization of the Study

Each participant completed nine training sessions over the 3 weeks. The sessions were performed three times per week with at least 1 day of rest in between. Session 1 involved six bouts, sessions 2 and 3 involved four bouts, sessions 4–6 involved five bouts, and sessions 7–9 involved six bouts. The average power produced during each of the total of 47 bouts was used in the analysis, and heart rate (HR) was measured during all training sessions. During the first and last training sessions, voluntary evoked knee extension forces were measured before and 5 min, 1 h, and 24 h after the exercise session. During the first and last training sessions, blood lactate concentration was measured before and 5 min and 1 h after the exercise session. Each participant completed a test to measure peak oxygen uptake (VO_2_peak) before and after training.

### 2.4. Familiarization and Warm-Up

A familiarization session was performed 7 days before the first training session. The participant was seated in the dynamometer chair (System 3; Biodex Medical Systems, Shirley, NY, USA) and performed several attempts of maximal knee extension. The participant then was familiarized with stationary cycling on a mechanically braked cycle ergometer (Monark 824E Monark, Vansbro, Sweden) by performing several submaximal and one maximal ~10 s cycling bouts against a brake weight of 3.75% of their body weight.

### 2.5. Cycling Exercise

A Monark 824E ergometer with the brake weight equal to 7.5% of the participant’s body weight was used for SIT training. Participants were instructed to remain in a seated position during all training sessions. Each active bout of exercise involved 30 s of all-out cycling with 4 min of rest pedaling against minimal resistance at a comfortable rate between bouts.

### 2.6. Measurements of HR and Lactate Concentration

HR was recorded in all training sessions and during both VO_2_peak tests using a HR monitor (S-625X; Polar Electro, Kempele, Finland). Peak HR values were averaged over consecutive 5 s intervals during each cycling bout and were used to calculate the average peak HR of each training session [10]. Maximal HR (HRmax) was defined as the highest 5 s averaged HR attained during the latest stages of the baseline VO_2_peak test.

Venous blood samples (5 mL) were collected at the baseline and then 5 min and 1 h after the last bout during the first and the last SIT sessions. Whole-blood lactate concentration was measured immediately using a portable lactate analyzer (Pro™ LT-1730; Arkray Inc., Kyoto, Japan).

### 2.7. Measurement of Torque

With the participant in a seated position, maximal voluntary contraction (MVC) isometric peak torque was measured for the knee extensor muscles on the dominant leg using an isokinetic dynamometer (System 3; Biodex Medical Systems). The procedure was previously described by Krusnauskas et al. (2018). Briefly, the participant sat in the dynamometer chair with the knee joint positioned at a 60° angle (0° = full knee extension). MVC was required to be produced and sustained for ~2 s in two attempts, and the larger of the two values was used in the analysis.

### 2.8. VO_2_peak Test

The VO_2_peak test was performed 1 week before the first training session and within 1 week after the last training session. Before testing, body height (Martin, GPM Siber Hegner, Switzerland) and body composition (Tanita-305 body composition monitor, Tanita Corp., Tokyo, Japan) were measured. Gas analysis was performed using a portable breath-by-breath analyzer (Oxygen Mobile; Jaeger/VIASYS Healthcare, Hoechberg, Germany). The protocol started with 3 min of cycling at 50 W, after which the load was increased by 5 W every 10 s. Participants were instructed to maintain a pedaling cadence of 60 rpm [10]. The test was continued until volitional fatigue, which was defined as an inability to sustain the required pedaling cadence. VO_2_peak was recorded as the highest VO_2_ rolling average over a 30 s period.

## 3. Statistics

The data are presented as mean ± SD. The normality of the data distribution was evaluated using the Kolmogorov–Smirnov and Shapiro–Wilk tests: all data were found to be normally distributed except for lactate concentrations. Repeated-measures analysis of variance (general linear model) and Tukey’s post hoc test were used to identify the within-factor effects (time: before, 5 min, 60 min, and 24 h after exercise or in bouts 1–6) for outcome measures (MVC and average power). The nonparametric Friedman’s test with Dunn’s post hoc test was used to identify the within-factor effects of time for lactate concentration (time: before, 5 min, and 60 min after exercise). Values were compared between groups at a single time point using the nonparametric Kruskal–Wallis test with Dunn’s post hoc test. Significance was set at *p* < 0.05. The data were analyzed using IBM SPSS Statistics for Windows (version 25.0; IBM Corp., Armonk, NY, USA).

## 4. Results

### 4.1. Anthropometric Data

The anthropometric data did not differ between the high and low responders (*p* > 0.05) (Table 1).

### 4.2. Power Production during Training

**Figure 1:**
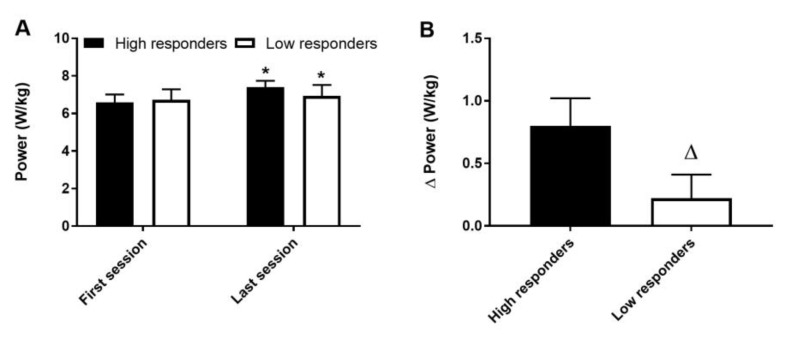
Average power produced (**A**) and difference in power between the first and last training sessions (**B**). * different from the first session (*p* < 0.05), Δ different between groups (*p* < 0.05). Data are expressed as mean and SD.

**Figure 2:**
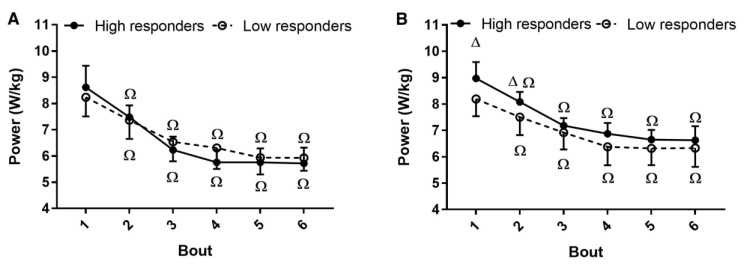
Power across the bouts of the first (**A**) and last (**B**) training sessions. Ω different from the first bout (*p* < 0.05); Δ different between groups (*p* < 0.05). Data are expressed as mean and SD.

Average power produced in the first training session did not differ between groups (*p* = 0.54). Although average power increased after training in both groups (*p* < 0.001) (Figure 1A), the increase was much higher in the high responders (*p* < 0.001) (Figure 1B). No interaction between group and bout for power produced was observed during the first (*p* = 0.15) or last (*p* = 0.53) training sessions (Figure 2A,B). However, a group effect was detected in the last session (*p* < 0.001) and a bout effect was detected in both the first (*p* < 0.001) and last (*p* < 0.001) sessions.

### 4.3. HR and Blood Lactate Concentration

**Figure 3:**
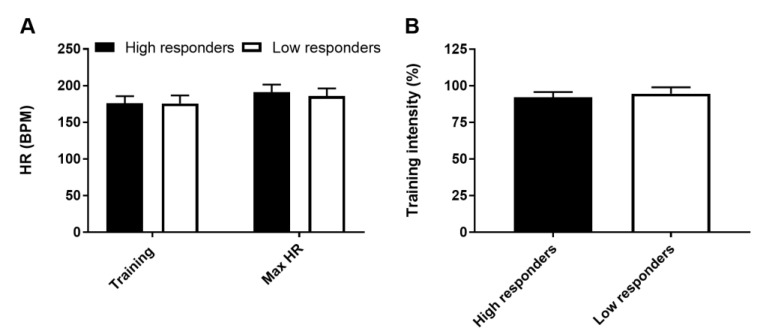
Peak heart rate (HR) during training and peak oxygen uptake (VO_2_peak) test (HRmax) (**A**) and averaged training peak HR as a percentage of HRmax (**B**). Data are expressed as mean and SD.

**Figure 4:**
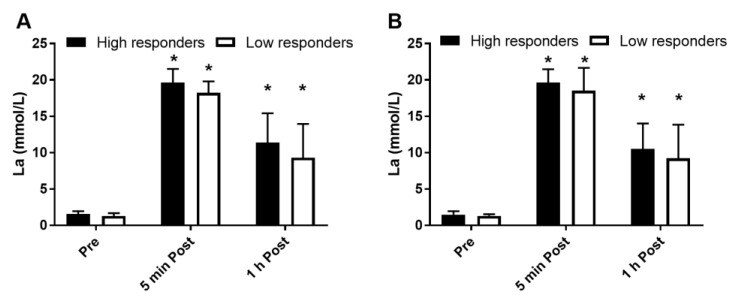
Blood lactate concentration at baseline (pre) and 5 min and 1 h after the first (**A**) and last (**B**) training sessions. * different from pre (*p* < 0.05). Data are expressed as mean and SD.

HRmax measured during the baseline VO_2_peak test did not differ significantly between high and low responders (*p* = 0.45) (Figure 3A). Training HR relative to HRmax also did not differ between groups (92.2 ± 3.6% in high vs. 94.6 ± 4.6% in low responders, *p* = 0.17), indicating that training was performed at a similar intensity in the two groups (Figure 3B). Baseline lactate concentrations did not differ between groups or between SIT sessions (*p* = 0.66). The blood lactate concentration increased significantly for up to 1 h after the exercise sessions and the magnitude of this increase did not differ between groups (Figure 4A,B). No main effects of group or test time, or interaction between these variables and training session were observed (*p* = 0.95).

### 4.4. MVC

**Figure 5:**
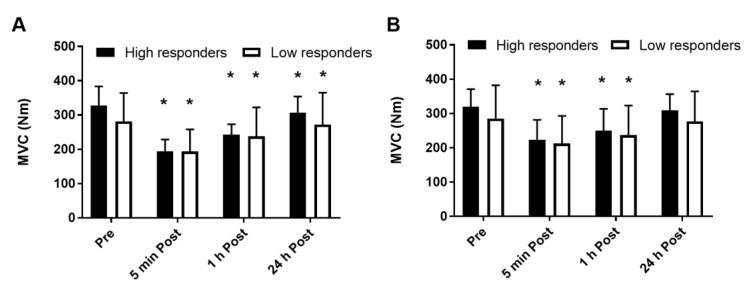
Maximal voluntary contraction (MVC) before (pre) and 5 min, 1 h, and 24 h after the first (**A**) and last (**B**) training sessions. * different from pre (*p* < 0.05). Data are expressed as mean and SD.

Both groups had a similar reduction in MVC after the first (Figure 5A) and last (Figure 5B) SIT sessions (*p* = 0.40). After the first session, MVC did not recover fully within 24 h. By contrast, MVC recovered fully within 24 h after the last SIT training session. No main effects of group or test time, or interaction between these variables and training session were observed (*p* > 0.10) for MVC.

### 4.5. Aerobic Capacity (VO_2_peak)

**Figure 6:**
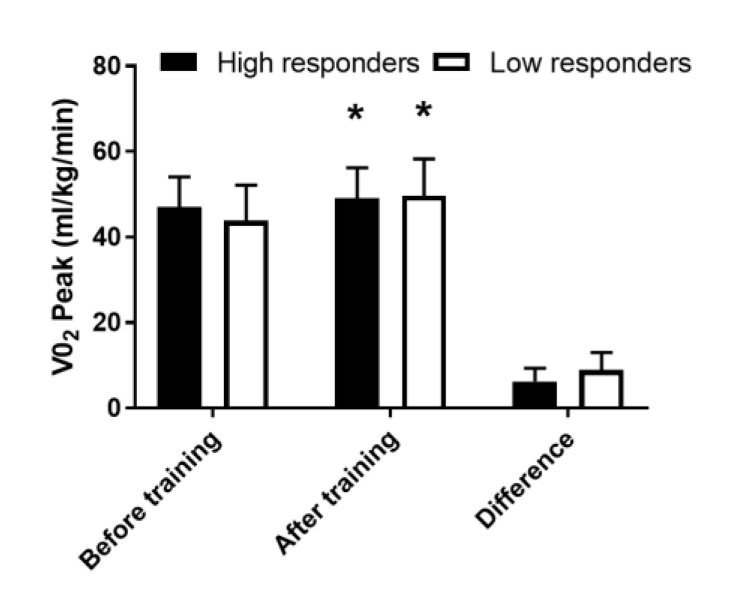
VO_2_peak before (pre) and after training. * different from pre (*p* < 0.05). Data are expressed as mean and SD.

VO_2_peak did not differ significantly between groups at the baseline (*p* = 0.63). Three weeks of SIT improved VO_2_peak (*p* = 0.03 compared with before training, *p* = 0.98 for the comparison between groups, Figure 6) independently of the change in repeated sprinting average power.

## 5. Discussion

The main findings of the current study are, first, that three weeks of SIT increased aerobic capacity to the same extent in high and low responders for averaged repeated sprinting power and, second, that the magnitude of the increase in repeated sprint cycling performance was independent of the physical exertion level during the training sessions. These results suggest that future studies should focus on assessment of other markers associated with adaptations to SIT or the improvement in aerobic capacity.

We had expected that SIT would improve aerobic capacity because it is known that low-volume HIIT induces adaptations that are both “peripheral” (increased skeletal muscle oxidative metabolism [19]) and “central” (augmented blood volume, stroke volume, and cardiac output [20,21]). Although SIT per se implies all-out effort, in reality, the level of exertion may differ between people. Such individual differences in exertion level could induce different stress levels, which hypothetically may affect the extent of adaptation. However, the results of the current study indicate that the high and low responders were training at a similar intensity and effort in the repeated sprint cycling. Given that the exercise duration was fixed and tightly controlled, we can rule out differences in the load parameters as a contributor to the differences in adaptation between the low and high responders. Moreover, given that the increase in VO_2_peak was similar between the high and low responders, it is unlikely that the aerobic component can explain the differences in responses between groups.

Adaptations to endurance training [22,23] and of anaerobic capacity to high-intensity training both have a significant genetic component in young healthy people [24]. Although skeletal muscle fiber type composition is genetically predetermined to a large extent [25], fibers of any type can undergo phenotypic adaptation under appropriate stimulation. The predominant involvement of and fatigue development in type II muscle fibers with increasing exercise intensity are well known [26,27]. Animal studies have shown that the faster the composition of the myosin heavy chain in muscle fibers, the more sensitive those fibers are to fatigue development [28,29]. It has recently been shown that muscle force recovery after SIT is much longer in people with predominantly type II muscle fiber composition [30]. There is evidence that oxidative reprogramming with training is much easier across a pool of type II fiber subtypes than that of type I fibers [31]. This suggests that those who developed a stronger response to the SIT intervention may have had a higher proportion of type II fibers, which would have made their muscles better suited for adaptation to this specific type of training. However, in the current study, we found that the decrease in average power across bouts did not differ between high and low responders even after three weeks of SIT, despite the quicker recovery of MVC after the training. We therefore suggest that the different responses to SIT may not reflect differences in fiber type composition.

Although there was a clear trend for a higher lactate concentration in the high responders during the first training session, the difference between groups was not significant. It is possible that high responders were able to maintain higher anaerobic energy turnover flux through both greater lactate production during the sprints and faster utilization during the recovery between sprints, which may have contributed to an improved exercise capacity. However, this is only a supposition because we did not assess the lactate kinetics during and immediately after exercise, which is a limitation of our study. It may also be speculated that the high responders became capable of using more of their increased aerobic potential during the SIT session as a result of the training. This ability is one of the key aspects of aerobic conditioning [32] and may also differentiate between more and less sports-talented individuals [24]. To examine this issue, expired gas analysis must be measured during SIT sessions, which we did not include in the current study.

Finally, the improvement in repeated sprint cycling performance may have been achieved by improved technique (skills) or movement economy. We did not analyze the cycling technique, but the same stationary mechanically braked cycle ergometer was used and the same individual configuration of the apparatus (seat height, handlebar position, brake weight) for each participant was ensured throughout all supervised training sessions. This reduced the potential for bias related to changes in the musculature involved (e.g., by ensuring that knee extension was performed mainly by the quadriceps muscles). The participants were instructed to always maintain the seated position on the bike (i.e., pedaling in the standing position was not allowed) to keep the movement pattern as simple and consistent as possible. However, to increase the stability of the feet on the pedals, the cycling sprints were always conducted with pedal straps, and mastering the “up” phase of the pedaling cycle to some extent, for example, by involving the knee flexors, cannot be excluded in some participants; that is, more of the responders may have used this technique.

Other technique-related factors also cannot be discounted as possible contributors to our results. These possible factors include more symmetrical distribution of the load between the legs, smoother concentric muscle contractions rather than an “impulsive” pedaling pattern, or involvement of the upper body musculature when supporting the body position by using the contralateral arm on the handlebar along with other technical aspects of cycling (e.g., pelvic tilt or foot actions, which allow for recruitment of different muscles or the same muscle at different lengths). This may be relevant if the responders had cycled less frequently in the past (regardless of the location or type of cycling) and would therefore be considered to have been more naïve to the exercise and to have had a greater capacity for improvement. Although the participants were not cyclists at the time of the experiment or in the past, we cannot be certain that the low responders did not have more experience using a stationary cycle ergometer in a gym or at home. However, if that were the case, we would probably have seen some baseline differences in cycling efficiency or performance between the groups, which we did not, and we believe that these speculations would have had little or minimal effect.

Possible confounding factors such as genetic, biomechanical, and lifestyle factors including other stressors, nutritional/dietary factors, and the duration and quality of sleep may have influenced the extent of the improvement in repeated sprint capacity after the SIT. However, assessment of such factors was not feasible and was outside the scope of the present study.

## 6. Conclusions

Our main conclusion is that the extent of the improvement in fatigue resistance in response to three weeks of SIT cannot be explained by the exertion level as assessed from the peak HR achieved during each of the sessions or decrements in lactate concentration and muscle force after the first and last sessions. Future studies are needed to examine other possible factors that could explain the high and low responses to SIT.

## Figures and Tables

**Table 1:** Characteristics of the participants.

	High Responders (*n* = 17)	Low Responders (*n* = 9)
Age (years)	25.9 ± 6.4	25.3 ± 5.4
Height (cm)	182.8 ± 7.3	183.8 ± 9.9
Weight (kg)	78.9 ± 11.1	84.5 ± 16.6
Body mass index (kg/m^2^)	23.5 ± 2.4	24.7 ± 3.2
Fat mass (%)	15.9 ± 3.6	16.3 ± 5.6
VO_2_peak (mL/kg/min)	47 ± 7	43.9 ± 8.2

Note: data expressed as mean and standard deviation (SD).
